# Neutrophil serine proteases in cystic fibrosis: role in disease pathogenesis and rationale as a therapeutic target

**DOI:** 10.1183/16000617.0001-2024

**Published:** 2024-09-18

**Authors:** Marcus A. Mall, Jane C. Davies, Scott H. Donaldson, Raksha Jain, James D. Chalmers, Michal Shteinberg

**Affiliations:** 1 Department of Pediatric Respiratory Medicine, Immunology and Critical Care Medicine and Cystic Fibrosis Center, Charité – Universitätsmedizin Berlin, corporate member of Freie Universität Berlin and Humboldt-Universität zu Berlin, Berlin, Germany; 2 German Center for Lung Research (DZL), associated partner site, Berlin, Germany; 3 Berlin Institute of Health at Charité – Universitätsmedizin Berlin, Berlin, Germany; 4 National Heart and Lung Institute, Imperial College London, London, UK; 5 Royal Brompton Hospital, Guy's and St. Thomas’ NHS Foundation Trust, London, UK; 6 Department of Medicine, Division of Pulmonary Diseases and Critical Care Medicine, The University of North Carolina at Chapel Hill, Chapel Hill, NC, USA; 7 Department of Internal Medicine, University of Texas Southwestern Medical Center, Dallas, TX, USA; 8 University of Dundee, Dundee, UK; 9 Lady Davis Carmel Medical Center, Haifa, Israel; 10 The B. Rappaport Faculty of Medicine, Technion Institute of Technology, Haifa, Israel

## Abstract

Chronic airway inflammation is a central feature in the pathogenesis of bronchiectasis (BE), which can be caused by cystic fibrosis (CFBE; hereafter referred to as CF lung disease) and non-CF-related conditions (NCFBE). Inflammation in both CF lung disease and NCFBE is predominantly driven by neutrophils, which release proinflammatory cytokines and granule proteins, including neutrophil serine proteases (NSPs). NSPs include neutrophil elastase, proteinase 3 and cathepsin G. An imbalance between NSPs and their antiproteases has been observed in people with CF lung disease and people with NCFBE. While the role of the protease/antiprotease imbalance is well established in both CF lung disease and NCFBE, effective therapies targeting NSPs are lacking. In recent years, the introduction of CF transmembrane conductance regulator (CFTR) modulator therapy has immensely improved outcomes in many people with CF (pwCF). Despite this, evidence suggests that airway inflammation persists, even in pwCF treated with CFTR modulator therapy. In this review, we summarise current data on neutrophilic inflammation in CF lung disease to assess whether neutrophilic inflammation and high, uncontrolled NSP levels play similar roles in CF lung disease and in NCFBE. We discuss similarities between the neutrophilic inflammatory profiles of people with CF lung disease and NCFBE, potentially supporting a similar therapeutic approach. Additionally, we present evidence suggesting that neutrophilic inflammation persists in pwCF treated with CFTR modulator therapy, at levels similar to those in people with NCFBE. Collectively, these findings highlight the ongoing need for new treatment strategies targeting neutrophilic inflammation in CF lung disease.

## Shareable abstract

**There is an ongoing need for new treatment strategies targeting neutrophilic inflammation in CF lung disease. The neutrophilic inflammatory profiles in CF lung disease and NCFBE are similar, potentially permitting a similar therapeutic approach.** https://bit.ly/3xYC4Hh

## Introduction

**FIGURE 1:**
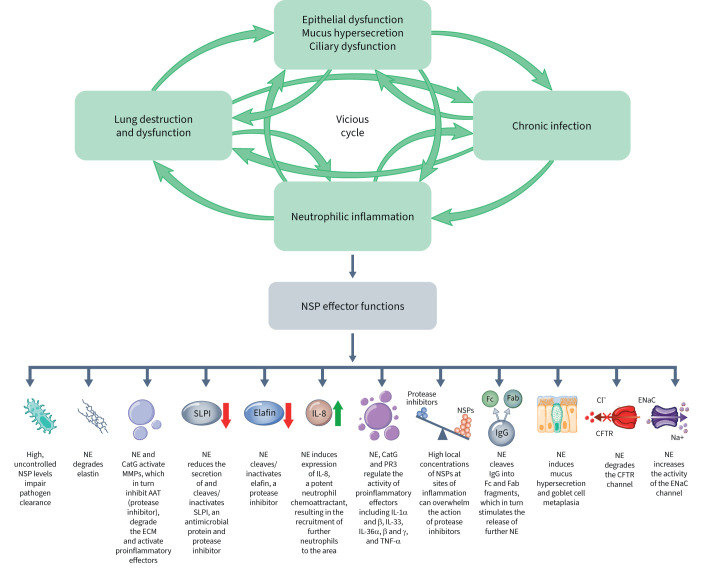
The role of neutrophil serine proteases (NSPs) in chronic inflammatory lung disease (cystic fibrosis (CF) lung disease and non-CF bronchiectasis). AAT: alpha-1 antitrypsin; CatG: cathepsin G; CFTR: CF transmembrane conductance regulator; Cl^−^: chloride; ECM: extracellular matrix; ENaC: epithelial sodium channel; Fab: antigen-binding fragment; Fc: crystallisable fragment; IgG: immunoglobulin G; IL: interleukin; MMP: matrix metalloproteinase; Na^+^: sodium; NE: neutrophil elastase; PR3: proteinase 3; SLPI: secretory leukocyte protease inhibitor; TNF: tumour necrosis factor.

Cystic fibrosis (CF) is a life-limiting genetic disease affecting the lungs and multiple other organs that is caused by pathogenic variants in the gene encoding the CF transmembrane conductance regulator (CFTR) protein [1, 2]. CFTR is an anion channel that transports chloride and bicarbonate ions across apical epithelial cell membranes and works in tandem with other ion channels, including the epithelial sodium channel (ENaC). Together, these channels maintain the homeostasis of airway surface liquid volume and pH, which is essential for the proper formation, hydration and viscoelasticity of airway mucus; this, in turn, is critical for effective mucociliary clearance (MCC), a primary defence mechanism of the lungs [3–7]. Loss of CFTR function in the CF airway epithelium leads to mucus hyperconcentration, increased mucus viscoelasticity and mucociliary dysfunction. The resulting mucus obstruction of the airways creates an environment that promotes infection and chronic inflammation, which feed forward to create a pathogenic “vicious cycle” [8]. In most people with CF (pwCF), chronic inflammation leads to the onset of bronchiectasis (BE), a chronic and progressive respiratory condition characterised by structural lung damage and irreversible dilatation of the airways [9]. Chronic neutrophilic inflammation is a central driver of BE and is associated with high, uncontrolled levels of neutrophil-derived serine proteases (NSPs), including neutrophil elastase (NE), cathepsin G (CatG) and proteinase 3 (PR3). These NSPs promote and maintain a pathogenic environment in BE (figure 1) [10]. As pwCF almost invariably develop diffuse and progressive BE [1], we hereafter use the term CF lung disease to describe CFBE, unless otherwise specified in the literature described in this review.

The burden of BE on healthcare systems around the world is increasing [11]. In CF, the introduction of CFTR modulator therapy, which targets the basic defect by restoring function of the defective CFTR protein at the apical cell membrane, has immensely improved CF lung disease for those with eligible *CFTR* mutations [7]. CFTR modulator therapies are increasing life expectancy, resulting in a larger population of older people with BE. However, evidence suggests that airway inflammation persists, even in pwCF treated with CFTR modulator therapy [12, 13]. Although elevated NE activity is well described in people with CF lung disease and people with non-CF BE (NCFBE) [10, 14], no effective anti-inflammatory therapies targeting NSPs have been licensed. In this review, we summarise the evidence for neutrophilic inflammation in CF lung disease in order to address whether neutrophilic inflammation and high, uncontrolled NSP levels play a similar role in CF lung disease in the era of CFTR modulator therapy.

## The production and function of NSPs

### Production, activation, storage and release of NSPs

NSPs are produced during neutrophil differentiation in the bone marrow [15]. They are synthesised as inactive precursors that require activation by cathepsin C (CatC; also known as dipeptidyl peptidase 1) [15]. Active NSPs are stored in azurophil granules within neutrophils [15]. Upon neutrophil activation at sites of inflammation, NSPs are secreted into the extracellular environment [16]. If antiproteases are present at sufficient amounts, such that the antiprotease shield (a defence system to dampen and control excessive protease activity) remains intact, free NSPs are inhibited by antiproteases in the extracellular milieu [17, 18]. However, a portion of the released NSPs remain bound to the neutrophil cell surface [16, 19–21]. These membrane-bound NSPs are catalytically active, but, unlike free NSPs, are remarkably resistant to antiproteases [19, 20]. As increased membrane-bound NE on activated neutrophils is protected from antiprotease inhibition [19], it can exert damaging effects even when free NE is still fully inhibited by an intact antiprotease shield. This is exemplified by a mouse model with CF-like lung disease that has no free NE, but has increased membrane-bound NE activity that is sufficient to cause structural lung damage [22, 23], as well as studies in pwCF showing that membrane-bound NE on sputum neutrophils correlates with lung disease severity [24].

### NSP effector functions

NSP effector functions are summarised in figure 1. High levels of NE result in elastin degradation [10] and high levels of both NE and CatG result in degradation of the extracellular matrix *via* activation of matrix metalloproteinases (MMPs); together, this leads to tissue remodelling and structural lung damage [25–27]. NE induces expression of interleukin (IL)-8, a potent neutrophil chemoattractant, in airway epithelial cells [28]. This creates a self-perpetuating cycle whereby NE released from neutrophils induces secretion of IL-8 from the airway epithelium, which in turn recruits additional neutrophils to the area [28]. In addition to upregulating the expression of IL-8, NE also regulates the activity of IL-1α, IL-1β, IL-33, IL-36α and IL-36γ [29], and increases the expression of tumour necrosis factor-α and IL-1β from macrophages [25], resulting in further neutrophil recruitment and airway inflammation. PR3 and CatG also play a role in the regulation of proinflammatory effectors IL-36α, β and γ, IL-1α and β, and IL-33 [29].

High local concentrations of NSPs at sites of inflammation can overwhelm the action of antiproteases [30]. Additionally, high levels of NE lead to the cleavage and inactivation [31], as well as the reduced secretion, of secretory leukoprotease inhibitor (SLPI) [32] and the cleavage of elafin [33], thus promoting a protease–antiprotease imbalance. NE-induced activation of MMPs inhibits another antiprotease, alpha 1 antitrypsin (AAT) [10], further disrupting the protease–antiprotease balance. NE also cleaves immunoglobulin G into Fc and Fab fragments, which in turn stimulates the release of further NE [34].

In addition to the effector functions mentioned above, NE has additional roles in different aspects of CF lung disease, including goblet cell metaplasia and mucus secretion, ion transport modulation, and host defence, which are reviewed below.

## Neutrophilic inflammation and NSPs in CF lung disease

### Role of neutrophilic inflammation and NE in established CF lung disease not treated with CFTR modulator therapy

PwCF show evidence of impaired MCC, airway mucus obstruction, chronic, neutrophil-predominant airway inflammation, infection and progressive structural lung damage [35, 36], with chronic neutrophilic inflammation being a central feature [36]. Under normal circumstances, the primary function of neutrophils is to remove pathogens through phagocytosis, after which they undergo apoptosis and are cleared by macrophages. Exaggerated neutrophil responses, as seen in CF lung disease, lead to extracellular release (exocytosis) of toxic neutrophil granule products and proinflammatory cytokines through degranulation and neutrophil extracellular trap (NET) formation. NE, CatG and PR3 are contained within the primary (azurophilic) granules of neutrophils and are released in high concentrations during chronic inflammation [9, 37]. Increased NSP activity further impairs MCC, drives mucus hypersecretion, causes structural lung damage and weakens host defences [38–40] (figure 1). Clinically, high levels of airway neutrophils, NE and NETs correlate with adverse outcomes, including low microbiome diversity and *Pseudomonas* (*P.*) *aeruginosa* infection, pulmonary exacerbations, hospitalisations and mortality [41–43]. Furthermore, sputum NE has been identified as an indicator of reduced lung function [44, 45]. In one study of pwCF aged 10 years and older, sputum NE had a strong negative correlation with percent predicted forced expiratory volume in 1 s (ppFEV_1_) [44, 45]. Sputum NE was also negatively correlated with ppFEV_1_ in a second study in pwCF (ages 9–54 years), and in a multivariable regression analysis, NE and neutrophil counts were able to explain the majority of variation in FEV_1_ [45].

### Role of NE and neutrophilic inflammation in onset and progression of early lung disease in CF

As described above, the role of neutrophilic inflammation in established CF lung disease has been relatively well characterised. However, its role in the onset and progression of CF lung disease, usually in childhood, is less well understood. Nevertheless, neutrophilic inflammation and high levels of NE are known to play a key role in younger populations. In a 2009 study in children with CF, Sly *et al.* [46] found that free NE activity was associated with the future development of structural lung disease even in the absence of current bacterial airway infection. Sly *et al.* [47] then went on to identify NE activity in bronchoalveolar lavage fluid (BALF) as a significant predictor of early BE in young children with CF; the prevalence of BE at each visit increased with age (up to 3 years of age). A subsequent study in children with CF showed that lung function decline was associated with increased neutrophil counts, IL-1β and free NE activity, and decreased NSP–antiprotease activity (NE–antiprotease complexes and SLPI) [48]. Of these inflammatory markers, the authors concluded that NE activity was the most informative biomarker to monitor disease activity [48]. In a study of early CF, BE was significantly associated with the frequency with which NE activity, but not infection, had been detected in BALF. This suggests that pulmonary inflammation has a cumulative effect on the extent of structural lung disease, possibly more so than infection [49]. Indeed, a study by Garratt *et al.* [50] in children with CF demonstrated that NE activity was significantly associated with BE progression. A recent study examined childhood factors associated with adolescent lung function; elevated BALF NE was associated with more rapid lung function decline, suggesting that action within the first 5 years of life to prevent and/or treat respiratory exacerbations and counteract lower airway neutrophilic inflammation may slow lung function decline in children with CF [51].

Some studies have shown neutrophilic inflammation in the absence of (or prior to) structural changes in the lung. A study by Esther *et al*. [52] of preschool children with CF (the AREST CF programme) revealed increased mucus burden and inflammatory markers in the absence of structural lung disease. In another study by Muhlebach *et al.* [53], children with CF who had low levels of airway-derived bacterial DNA presented with inflammation but no significant structural lung damage. These findings are supported by a subsequent study in CF infants (1 year of age) in which only a weak association between inflammation, abnormal physiology and structural changes was found [54]. Additionally, in a study in infants and preschool children with CF aged 3–62 months, high NE exocytosis by airway neutrophils correlated with BE and lung damage, whereas the molecular measure of free extracellular NE activity did not [55]. This finding is consistent with studies in a mouse model of CF lung disease showing that increased membrane-bound NE on the neutrophil surface is present before “free” NE activity becomes detectable [22], probably because the antiprotease shield remains intact in early lung disease, and that elevated membrane-bound NE alone is sufficient to cause structural lung damage. Taken together, these data suggest that airway NE activity is a key driver in the onset and progression of lung disease in children with CF.

Overall, evidence indicates that high sputum NE activity, resulting from a protease–antiprotease imbalance, is the key component of neutrophilic inflammation that drives the onset and progression of lung disease, specifically BE, in adults and children with CF.

## Role of NSPs in different aspects of CF lung disease

Studies demonstrate that neutrophilic inflammation and chronically elevated NSP activity play key roles in the pathophysiology of CF lung disease.

### Goblet cell metaplasia and mucus hypersecretion

A study by Voynow *et al.* [56] in murine airways showed that NE induces expression of the protein mucin 5AC, oligomeric mucus/gel-forming (MUC5AC), a secreted mucin and one of the key components of airway mucus. NE also causes goblet cell metaplasia (goblet cells are not typically found in murine airways) (figure 1). These findings are corroborated in a study by Gehrig *et al.* [22], in which elevated mucin expression (*Muc5ac*, *Muc5b* and *Gob5*) and goblet cell metaplasia were significantly reduced in NE knockout mice with CF-like disease. However, the absence of NE did not lead to a reduction in mucus plugging [22]; this may be because expression of *Muc5ac*, *Muc5b* and *Gob5*, although decreased, remained at near-normal levels observed in wild-type mice, suggesting that constitutive mucus secretion remained intact [22]. Further, due to the hyperabsorptive ion transport phenotype of the mouse model, the constitutively secreted mucus was hyperconcentrated [22]. Given that an increased mucus concentration causes impaired MCC [57, 58], this likely explains the persistent mucus plugging in the NE knockout mice with CF-like disease, despite the reduction in goblet cell metaplasia and mucin expression. NE also significantly increased keratinocyte-derived chemokine and IL-5 in BALF and increased lung tissue inflammation and BALF leukocyte counts, suggesting that NE proteolytic activity initiates an inflammatory process leading to goblet cell metaplasia [56]. A further study by Fischer *et al.* [39] in human bronchial epithelial cells demonstrated that the oxygen radical scavenger, dimethylthiourea, inhibited NE-induced increases in MUC5AC expression. NE also increased cellular fluorescence in these cells, indicating the generation of intracellular reactive oxygen species, supporting the hypothesis that NE increases *MUC5AC* gene expression by an oxidant-dependent mechanism. PR3 also plays a role in mucus hypersecretion, as seen in a study by Witko-Sarsat *et al.* [59], where PR3 purified from neutrophil azurophil granules triggered airway gland secretion. Collectively, these studies indicate a key role of NE and PR3 in the mechanism of mucus hypersecretion, which aggravates mucus hyperconcentration and mucociliary dysfunction in CF.

### Ion transport modulation

The CFTR and ENaC proteins are ion channels that, together, are key to the coordinated secretion and absorption of salt and water across airway surfaces, and hence maintenance of airway surface liquid homeostasis. A study of a murine *P. aeruginosa* lung infection model demonstrated an association between NE and CFTR degradation (figure 1), thus establishing a link between NE activity and loss of CFTR function in bacterial lung infections relevant to CF [60]; this could lead to impairment in airway ion transport in other chronic inflammatory lung diseases in which CFTR has been implicated, such as COPD [61].

NE also increases the activity of ENaC (figure 1) by proteolytic cleavage of the channel, resulting in sodium ion/fluid hyperabsorption, reduced airway surface liquid height and dehydrated mucus, culminating in inefficient MCC, thus playing a key role in the pathogenesis and progression of CF lung disease [62, 63]. In addition to the above work by Caldwell *et al.* [62] demonstrating activation of ENaC by NE, a study by Reihill *et al.* [64] showed that high levels of serine trypsin-like proteases (a different class of proteases to NSPs) are associated with poor lung function and survival in adults with CF, which may be the result of ENaC activation and airway dehydration. Together, these studies suggest a direct role of proteases, including NE, in the degradation of CFTR and activation of ENaC, impairing ion transport, leading to airway surface liquid dehydration and thus mucociliary dysfunction.

### Infection

In a study of pwCF, excessive proteolytic cleavage of C-X-C motif chemokine receptor 1 (CXCR1) induced the release of glycosylated CXCR1 fragments capable of stimulating IL-8 production in bronchial epithelial cells *via* Toll-like receptor 2, leading to a reduction in bacterial killing [65]. Inhalation of antiprotease alpha-1 antitrypsin restored CXCR1 expression and improved bacterial killing in individuals with CF. This demonstrates that an environment of high protease activity, such as chronic raised NE activity, perpetuates infection in CF.

## Impact of NSPs on endogenous antiproteases in CF lung disease

The major endogenous inhibitors of NSPs are AAT and the anti-leukoprotease superfamily enzymes SLPI and elafin [10, 30]. Under normal circumstances, these antiproteases keep the extracellular activity of NE, CatG and PR3 at a constant low level; however, high local concentrations of NSPs at sites of inflammation can overwhelm the action of antiproteases [30]. In a study in children with CF, despite normal concentrations of AAT and SLPI in the respiratory epithelial lining fluid (ELF), the majority of children had neutrophil-dominated inflammation and active NE present [66]. However, the majority of AAT and SLPI molecules were shown to be complexed and/or degraded [66], demonstrating a functional antiprotease deficiency due to the overwhelming neutrophil and NE burden [67]. Further, NE has been shown to result in the inactivation of antiproteases: in pwCF, high, uncontrolled levels of NE activity result in cleavage of SLPI [31]. Reduced levels of SLPI have been significantly associated with accelerated lung function decline in pwCF [14]. Additionally, elafin is cleaved by NE when present at excessive concentrations in CF sputum, and inhibition of NE is able to prevent the degradation of elafin [33]. NE also plays a role in AAT inhibition through activation of MMP-9 [68], which in turn inactivates AAT [69, 70].

Interestingly, AAT has been shown to have a direct effect on CFTR, with a study showing that AAT upregulated CFTR in primary bronchial epithelial air–liquid interface cultures, both at baseline and in the presence of inflammatory stimuli [71]. It can therefore be hypothesised that NE-mediated degradation of AAT could potentially lead to the further reduction of CFTR activity in pwCF. This finding may have implications in pwCF with residual CFTR activity and in pwCF treated with CFTR modulators.

## Emerging role of membrane-bound activity of NE and CatG in CF lung disease

In recent years, other NSPs have potentially been implicated in CF lung disease. NE activity has been shown to be elevated on the surface of airway neutrophils from mice with CF-like lung disease and neutrophil-bound NE has been shown to induce structural lung damage in these mice even in the absence of free NE activity [22]. Additionally, increased surface-bound NE has been identified on neutrophils sampled from the airways of pwCF; the activity of neutrophil-bound NE correlates with severity of CF lung disease, suggesting that this activity may play an important role in pathogenesis and serve as a novel biomarker [24, 72]. Additionally, CatG has been implicated in inflamed CF and COPD airways and may be involved in the pathogenesis of these conditions, thus representing a potential drug target [73]. CatG activity was also found to cause dysfunction of ciliated cells and destruction of airway epithelium in patients with BE and activity correlated with disease severity [74]. These studies suggest a role of membrane-bound NE and CatG in CF lung disease.

## Emerging links between mucus plugging, neutrophilic inflammation and structural lung damage in CF lung disease in the absence of bacterial infection

Although neutrophilic inflammation and structural lung damage in CF are closely linked to infection, they can also occur in its absence. Several studies have identified pathways and molecules that may be involved and, again, NE has been implicated. In children with CF, NE activity can be elevated in the absence of detectable bacterial infection [46] and can persist following successful *P. aeruginosa* eradication [50]. Evidence suggests that hypoxic epithelial necrosis may play an important role in the pathogenesis of neutrophilic inflammation, independent of bacterial infection, and identifies IL-1R as a novel target for anti-inflammatory therapy in CF [75]. In another study, IL-1α and IL-1β were detectable in BALF in the absence of infection, increased in the presence of bacterial infection and correlated with IL-8, neutrophil count and NE activity. In the same study, IL-1α was found to be associated with early structural lung damage in CF, implicating this pathway as a novel anti-inflammatory target [76]. Collectively, these findings suggest that inhibiting NE activity may benefit people with CF lung disease both in the presence and absence of infection.

## Inflammation, infection and NSP activity in pwCF receiving CFTR modulator therapy

CFTR modulator therapy has delivered unprecedented improvements in lung function, quality of life and pulmonary exacerbation frequency, even in people with advanced CF lung disease [77–81]. However, not all patients with CF are eligible for CFTR modulator therapy. Additionally, although some studies have demonstrated a reduction in airway inflammation and infection with CFTR modulators, other studies have reported substantial residual inflammation and infection, at least to levels similar to those observed in NCFBE [12, 13, 81, 82].

In a study of the CFTR modulator therapy ivacaftor in pwCF with the *G551D* gating variant, computed tomography scans revealed decreased airway mucus plugging up to 1 year after ivacaftor treatment [82]. Additionally, the concentrations of sputum inflammatory cytokines IL-1β and IL-8, and NE decreased significantly in the first week of treatment and continued to decline over 2 years. Rapid decreases in sputum *P. aeruginosa* density were also observed, although these rebounded after the first year of treatment [82]. In contrast, another study in people with the *G551D* variant showed that 6 months of ivacaftor treatment was not associated with significant changes in markers of inflammation (AAT, IL-1β, IL-6, IL-8, SLPI and NE) or airway microbial communities [83]. The findings between these two studies [82, 83] may differ due to differences in study design and infection status.

In two studies of the triple-combination CFTR modulator therapy elexacaftor/tezacaftor/ivacaftor (ETI) in pwCF with at least one F508del allele, restoration of CFTR function by ETI improved chronic airway infection and inflammation [12, 13]. In the first study, treatment resulted in decreases in free NE activity, CatG and PR3 in CF sputum over the first 12 months of therapy, but without reaching levels close to those seen in the healthy airway [13]. In the second study, treatment with ETI had a significant impact on airway inflammation, reduced protease levels, restored antiprotease levels and reduced sputum production (those with less severe disease being less likely to expectorate sputum at 3 months’ follow-up). However, at 12 months, levels of NE activity, PR3, CatG, IL-1β and airway neutrophils were not different from a cohort of people with NCFBE [12], confirming residual neutrophilic inflammation on ETI.

With regards to infection, treatment with ETI resulted in a shift of the CF sputum proteome towards that seen in healthy subjects, with improvements in microbiome α-diversity and decreases in the relative abundance of *P. aeruginosa* [13]. Additionally, a study of ETI found large and rapid reductions in traditional CF pathogens in sputum, whereas the burden of bacteria not typically considered as CF pathogens was unchanged. However, treatment with ETI did not result in complete eradication of CF pathogens in most participants [81].

The above studies suggest that residual inflammation and infection persist in pwCF treated with CFTR modulators (ivacaftor and ETI). These pwCF, and individuals with *CFTR* variants that are ineligible for or intolerant to CFTR modulator therapy (an estimated 10% of the global CF population), may therefore benefit from treatments that reduce neutrophilic inflammation, such as inhibition of NSP activity, as an approach distinct from the restoration of airway ion transport.

## Neutrophilic inflammation and high, uncontrolled levels of NSPs: CF lung disease *versus* NCFBE

The initiating mechanisms in CF lung disease and NCFBE are different. While the “culprit” event may be heterogeneous among the various aetiologies of NCFBE and is uncertain in many people (“idiopathic”) [84–86], CF lung disease originates from dysfunctional or absent CFTR protein at the airway epithelium. Despite the different initiating mechanisms, people with CF lung disease and people with NCFBE show evidence of neutrophilic inflammation. Neutrophilic inflammation of the airways is a central feature of NCFBE, the extent of which is associated with disease severity and progression [87, 88]. An imbalance between NSPs and their antiproteases is also well described in people with NCFBE, leading to high levels of free NSP activity in the airway [10].

NE has been shown to play a similar role in the pathobiology of both CF lung disease and NCFBE. As shown in CF, sputum NE activity has been associated with disease severity, an increased risk and frequency of exacerbations, infections, hospitalisations and mortality in adults with NCFBE [10]. In children with NCFBE, sputum NE correlated with exacerbations and disease severity [89]. Elevated sputum NE levels have been found to be associated with a decline in lung function (negatively correlated with FEV_1_) in adults with both CF and NCFBE. In the absence of CFTR modulator therapy, NE activity is, on average, higher in CF than in NCFBE, leading to earlier onset and mortality due to lung disease in CF [65].

In pwCF, reduced SLPI levels have been associated with accelerated lung function decline. Similarly, reduced SLPI has been correlated with reduced lung function and disease severity, as well as a shorter time to the next exacerbation, in people with NCFBE [90]. As already indicated, NE-mediated degradation of AAT could potentially lead to a further reduction of CFTR activity in pwCF with residual CFTR activity and those treated with CFTR modulators. Similarly, NE-mediated inactivation of AAT (through activation of MMP-9 [68], which in turn inactivates AAT [69, 70]) in NCFBE and the potential subsequent reduction of CFTR activity may compound BE by dehydrating the airway surface liquid [91].

There is more evidence for the role of NSPs, other than NE, in the pathobiology of NCFBE than in CF lung disease. In NCFBE, levels of PR3 were raised during exacerbations compared with stable state, and PR3 concentration was positively correlated with levels of sputum NE [92]. CatG activity has been found to cause dysfunction of ciliated cells and destruction of airway epithelium in patients with BE [74]. Additionally, increased CatG activity has been observed in people with NCFBE compared with healthy controls, with CatG activity increasing with increasing disease severity [74]. The roles of PR3 and CatG in the pathogenesis of CF lung disease need to be addressed in future studies.

Similar to the case in CF as described above, in NCFBE, neutrophilic inflammation can persist in the absence of infection and, in many cases, antibiotic treatment is insufficient to control infection [43]. Together, these similarities indicate that therapies inhibiting protease activity and restoring protease–antiprotease balance in the airways may benefit both people with CF lung disease and people with NCFBE through the inhibition of multiple NSPs.

## Previous and current NSP inhibitors in development

### AAT augmentation

Given the major role of AAT in the inhibition of NE, and the functional deficiency of AAT in CF lung disease due to the overwhelming neutrophil and NE burden, several studies have investigated the efficacy of AAT augmentation in pwCF [67]. Initial attempts *via* intravenous administration showed that high doses and repeat administration would be needed to achieve beneficial long-term effects [93]. As a result, aerosolised delivery of AAT was investigated in pwCF; this allows for delivery to the site of active airway disease while limiting systemic exposure and the need for high doses and intravenous access [67, 93, 94]. Contradictory results have been obtained, with some studies showing inhibition of NE in ELF [95, 96] and sputum [97], and others, including a randomised, placebo-controlled trial, showing limited effects on sputum NE activity and other inflammatory markers [98, 99]. These differences may reflect the use of different aerosol devices as well as sample types (ELF *versus* sputum) [67].

### NE inhibitors

To date, no drug has been licensed for the treatment of neutrophilic inflammation in CF lung disease or NCFBE. For the reasons described above, effective blockade of NSPs could have clinical benefits. Previous attempts at clinical development of NE inhibitors indicated for NCFBE and/or CF have not proceeded past phase II. AZD9668 (alvelestat) is an oral NE inhibitor that reached phase II, placebo-controlled testing in patients with BE [100]. Although AZD9668 improved lung function and there were trends for reductions in sputum inflammatory biomarkers, no change in sputum neutrophils was reported and no statistically significant difference in NE activity in sputum was reported between groups [100]. BAY 85-8501 is another oral NE inhibitor that reached phase IIa in patients with NCFBE [101]. NE activity in blood decreased significantly with BAY 85-8501; however, no changes in sputum NE activity, pulmonary function parameters or health-related quality of life were reported [101]. Both of these oral NE inhibitors failed to reduce sputum NE, indicating a lack of target engagement in the lung, and no further clinical trials in BE are ongoing. Several inhaled NE inhibitors have also been investigated. In a phase IIa clinical trial in adults with CF, DX-890 (EPI-hNE4) was well tolerated and the expected pharmacological effect on inhibition of NE in the lungs was observed [102]. However, no further clinical trials have been conducted since 2002. The inhaled NE inhibitor POL6014 (lonodelestat) reached phase II in patients with CF [103]. Inhibition of active NE in sputum was observed after a single administration but there have been no further publications since the phase I/II results were published in 2020. Lastly, a phase II trial of the inhaled NE inhibitor CHF6333 in both patients with CF and NCFBE was started in 2019 with recruitment completed, but no results have been published to date [104].

### CatC inhibitors

The compounds described thus far were designed to selectively inhibit NE activity; however, because multiple NSPs (NE, CatG and PR3) may play a role in neutrophilic inflammation, an approach that inhibits the activation of all three NSPs has the potential to be more successful. As CatC activates all NSPs during neutrophil development in the bone marrow [105], it is therefore an attractive target to reduce NSP activity. Furthermore, membrane-bound NE is remarkably resistant to endogenous inhibitors [19] and therefore potentially to extracellularly added therapeutic inhibitors. As such, an inhibitor of free NE activity may not be able to inhibit membrane-bound NE activity, in which case anti-NE strategies for extracellularly applied approaches may indeed need to be different. In contrast, CatC inhibition would target both free and membrane-bound NE, thus providing an important advantage over extracellularly added inhibitors, in addition to its broad targeting of all NSPs.

There are currently three oral CatC inhibitors (also known as dipeptidyl peptidase 1 inhibitors) in development, namely brensocatib, BI 1291583 and HSK31858 (table 1). In a phase I study, brensocatib was generally well tolerated in healthy subjects and indirectly inhibited NE activity *via* CatC inhibition during myelopoiesis in the bone marrow in an exposure-dependent manner [106]. Several dose-dependent, possibly CatC inhibition-related, nonserious skin findings were observed but did not prevent further clinical development [106]. As such, brensocatib has been tested in a 24-week phase II trial in people with NCFBE and was found to reduce sputum NE activity and significantly prolong the time to first exacerbation and lower the risk of exacerbation compared with placebo [107, 108]. Brensocatib has also been assessed in a 4-week phase II trial in pwCF; brensocatib was generally well tolerated irrespective of CFTR modulator status and exposure was comparable in pwCF and people with NCFBE [109, 110]. In phase I studies, BI 1291583 was found to be safe and well tolerated in healthy subjects, was readily absorbed and pharmacokinetics were supra-proportional [111]. The incidence of overall skin-related adverse events and drug-related skin exfoliation was similar between placebo and BI 1291583-treated subjects. BI 1291583 is currently being investigated in a 24–48-week phase II trial (Airleaf), which will assess efficacy, safety and dosing in people with NCFBE [112]; it is also being assessed in a 12-week phase II profiling study (Clairafly) evaluating safety, pharmacokinetics and pharmacodynamics in people with CF lung disease to inform the design of phase III trials [113, 114]. A phase II rollover study (Clairleaf) has also been initiated assessing long-term safety and efficacy of BI 1291583 [115]. HSK31858 is currently being investigated in a phase II trial assessing efficacy and safety in people with NCFBE [116]. Results from a phase I study of HSK31858 are not publicly available [117].

**TABLE 1:** Previous and current neutrophil serine protease (NSP) inhibitors in development for non-cystic fibrosis bronchiectasis (NCFBE) and/or cystic fibrosis (CF)

Inhibitor	Route of administration	Sponsor	Results summary
**NE inhibitors**			
AZD9668 (alvelestat)	Oral	AstraZeneca	Phase II in NCFBE [100]: Improved lung function Trends for reductions in sputum inflammatory biomarkers No change in sputum neutrophils No change in NE activity
BAY 85-8501	Oral	Bayer	Phase IIa in NCFBE [101]: Significant decreases in NE activity in blood No changes in pulmonary function parameters and health-related quality of life
DX-890 (EPI-hNE4)	Inhaled	Dyax and Debiopharm	Phase IIa in CF [102]: NE inhibition in the lungs was observed Well tolerated
POL6014 (lonodelestat)	Inhaled	Santhera Pharmaceuticals	Phase II in CF [103]: Inhibition of active NE in sputum after single administration
CHF6333	Inhaled	Chiesi Farmaceutici	Phase II in CF and NCFBE [104]: No results published to date
**CatC inhibitors**			
Brensocatib (INS-1007, AZD-7986)	Oral	Insmed Incorporated	Phase I [106]: Brensocatib was generally well tolerated in healthy subjects Inhibited NE activity in an exposure-dependent, indirect manner Several dose-dependent, possibly CatC-related, nonserious skin findings were observed^#^ Phase II in NCFBE [107, 108]: Significantly prolonged time to first exacerbation *versus* placebo in patients with NCFBE Lowered risk of exacerbations compared with placebo in patients with NCFBE Reduced sputum NE, CatG and PR3 activity Phase II in CF [109, 110]: Brensocatib exposure was comparable among participants with CF and NCFBE Brensocatib was generally well tolerated, irrespective of CFTR modulator status Phase III [120]: Currently being investigated in a phase III study (ASPEN) assessing the efficacy, safety and tolerability of brensocatib in participants with NCFBE
HSK31858	Oral	Haisco Pharmaceutical Group Co., Ltd.	Phase I [117]: Assessed the safety, tolerability, PK, PD and food effect of HSK31858 in healthy volunteers Results not yet publicly available Phase II [116]: Currently being investigated in a phase II trial assessing the efficacy and safety of HSK31858 in people with NCFBE
BI 1291583	Oral	Boehringer Ingelheim	Phase I [111]: BI 1291583 was safe and well tolerated in healthy subjects Most AEs were mild/moderate in intensity No serious AEs, AEs of special interest or deaths were reported BI 1291583 was readily absorbed and PK were supra-proportional Phase II [113–115]: Currently being investigated in a phase II study (Airleaf) assessing the efficacy, safety and dosing of BI 1291583 in participants with NCFBE Currently being investigated in a phase II profiling study (Clairafly) assessing the safety, PK and PD of BI 1291583 in participants with CF lung disease to inform the design of phase III trials Participants who have completed the Airleaf trial can be enrolled in a phase II rollover trial (Clairleaf) that will assess the long-term efficacy and safety of BI 1291583

^#^: these did not prevent further clinical development.

AE: adverse event; BE: bronchiectasis; CatC: cathepsin C; CatG: cathepsin G; CFTR: CF transmembrane conductance regulator; NE: neutrophil elastase; PD: pharmacodynamics; PK: pharmacokinetics; PR3: proteinase 3.

### Lessons from loss-of-function mutations in the *CatC* gene

Papillon–Lefèvre syndrome is a rare genetic disorder caused by loss-of-function mutations in the *CatC*/*DPP1* gene [118] that can provide useful considerations for the development of therapeutic CatC inhibitors. Firstly, people with Papillon–Lefèvre syndrome do not experience severe immunodeficiency [119]; therefore, severe immunodeficiency is not expected to occur in individuals receiving therapeutic CatC inhibitors. Secondly, people with Papillon–Lefèvre syndrome typically experience hyperkeratosis and periodontal disease [118]; these events have been observed in clinical trials of CatC inhibitors in healthy controls and people with BE, though they have not prevented clinical development and will continue to be monitored in future trials. Thirdly, pharmacological CatC inhibition reduces the activity of NSPs rather than inactivating them entirely, as occurs in patients with Papillon–Lefèvre syndrome. Although this may improve safety outcomes, partial inhibition of CatC may present challenges when treating patients with highly elevated free protease activity, as is observed in pwCF.

## Conclusion

The neutrophilic inflammatory profiles of people with CF lung disease and people with NCFBE share several similarities, potentially permitting a similar therapeutic approach. Studies comparing residual inflammation in pwCF on ETI *versus* healthy individuals [13] and people with NCFBE [12] indicate that there is residual inflammation in pwCF on CFTR modulator therapy, with biomarker levels similar to people with NCFBE. Similarly, *Pseudomonas* infection and airway dysbiosis persist in pwCF on CFTR modulator therapy, providing a strong stimulus for neutrophilic inflammation. Together, these findings highlight that even in the CFTR modulator therapy era, there is an ongoing need for new treatment strategies targeting both neutrophilic inflammation and infection in CF lung disease. In addition to inhibition of proteolysis implicated in the development of BE, inhibition of NSPs may have additional benefits in reducing mucus hypersecretion, improving airway ion transport and host defence in pwCF. Future studies on the effect of NSP inhibition in pwCF are warranted. It should be noted that due to low patient numbers, clinical development of treatments targeting neutrophilic inflammation may not be feasible for CF alone. However, as people with lung disease share a similar neutrophilic inflammatory profile with people with NCFBE, it should be possible to investigate both groups together. Outcomes from such a combined trial could inform on a potential therapeutic approach for BE, irrespective of aetiology.

**Questions for future research**

- Exploring the effect of NSP inhibition in people with CF, both treated and untreated with CFTR modulators.
